# Transcriptome profiling of pepper leaves by RNA-Seq during an incompatible and a compatible pepper-tobamovirus interaction

**DOI:** 10.1038/s41598-021-00002-5

**Published:** 2021-10-19

**Authors:** Balázs Kalapos, Csilla Juhász, Eszter Balogh, Gábor Kocsy, István Tóbiás, Gábor Gullner

**Affiliations:** 1 Agricultural Institute, Centre for Agricultural Research, Eötvös Lóránt Research Network (ELKH), Brunszvik utca 2, Martonvásár, 2462 Hungary; 2 Plant Protection Institute, Centre for Agricultural Research, Eötvös Lóránt Research Network (ELKH), Herman Ottó út 15, Budapest, 1022 Hungary

**Keywords:** Plant immunity, Plant stress responses

## Abstract

Upon virus infections, the rapid and comprehensive transcriptional reprogramming in host plant cells is critical to ward off virus attack. To uncover genes and defense pathways that are associated with virus resistance, we carried out the transcriptome-wide Illumina RNA-Seq analysis of pepper leaves harboring the *L*^*3*^ resistance gene at 4, 8, 24 and 48 h post-inoculation (hpi) with two tobamoviruses. *Obuda pepper virus* (ObPV) inoculation led to hypersensitive reaction (incompatible interaction), while *Pepper mild mottle virus* (PMMoV) inoculation resulted in a systemic infection without visible symptoms (compatible interaction). ObPV induced robust changes in the pepper transcriptome, whereas PMMoV showed much weaker effects. ObPV markedly suppressed genes related to photosynthesis, carbon fixation and photorespiration. On the other hand, genes associated with energy producing pathways, immune receptors, signaling cascades, transcription factors, pathogenesis-related proteins, enzymes of terpenoid biosynthesis and ethylene metabolism as well as glutathione S-transferases were markedly activated by ObPV. Genes related to photosynthesis and carbon fixation were slightly suppressed also by PMMoV. However, PMMoV did not influence significantly the disease signaling and defense pathways. RNA-Seq results were validated by real-time qPCR for ten pepper genes. Our findings provide a deeper insight into defense mechanisms underlying tobamovirus resistance in pepper.

## Introduction

Pepper (*Capsicum annuum* L.) is susceptible to infections by several viruses belonging to the genus *Tobamovirus* that afflict pepper production worldwide. Pepper-infecting tobamoviruses comprise *Bell pepper mottle virus* (BPeMV), *Obuda pepper virus* (ObPV), *Paprika mild mottle virus* (PaMMV), *Pepper mild mottle virus* (PMMoV), *Tobacco mild green mosaic virus* (TMGMV), *Tobacco mosaic virus* (TMV) and *Tomato mosaic virus* (ToMV)^1–6^. The most widely known, representative tobamovirus is TMV that has long served as a model to investigate the mechanisms of virus multiplication and interactions with host cells^7,8^. Tobamoviruses are very persistent in the environment (in crop debris or soil) and they are transmitted to healthy plants usually by mechanical wounds. Seed borne infection with tobamoviruses also occurs in pepper^4,8,9^. The genome of tobamoviruses consists of one single-stranded positive-sense RNA, 6.3–6.6 kb in size. This short RNA genome encodes at least four proteins: two replication proteins with partly overlapping sequences (126 and 183 kDa), a movement protein (MP, 30 kDa), and a coat protein (CP, 17.6 kDa)^10^.

Viruses are generally recognized by intracellular resistance proteins (R-proteins) in plant cells. In pepper, the resistance against tobamoviruses is conferred by different alleles of the *L* gene (*L*^*1*^, *L*^*2*^, *L*^*3*^ and *L*^*4*^), which encode CC-NB-LRR-type R-proteins that are highly homologous to each other (97.5%–98.9% identity)^11,12^. The pepper *L*^*3*^ gene is very efficient against most tobamoviruses except for some closely related isolates of PMMoV, which are able to overcome this type of resistance^3,13^. PMMoV is one of the most serious viral pathogen of pepper. Initially PMMoV infection causes only mild chlorotic or no foliar symptoms but the fruits usually show evident disease symptoms, which can cause considerable economic losses^14–16^. The *L*^*3*^ gene-mediated resistance is elicited by the PMMoV CP^15^. Another important tobamovirus, ObPV, sparked a lot of interest after its first isolation owing to its unique ability of overcoming the *N*-gene mediated resistance in tobacco^17,18^. The genomic organization of ObPV is similar to that of other tobamoviruses. Interestingly, the mutation of a single nucleotide in the 126 kDa gene of ObPV resulted in a virus clone that induced hypersensitive reaction (HR) in Xanthi NN tobacco^19,20^. However, in contrast to several PMMoV strains causing systemic infection, ObPV can not break the *L*^*3*^ gene-mediated resistance in pepper and elicits HR^2^.

Upon virus recognition by R-proteins, signals are transmitted to the nucleus in several temporal waves leading to the rapid and extensive reprogramming of gene expression patterns in host cells^21^. This reprogramming is controlled by a complex, multilayered regulatory network, in which defense-related plant hormones including salicylic acid, jasmonic acid and ethylene as well as various transcription factor proteins located in the nucleus play critical roles^22,23^. Resistance is determined by the timely recognition of the pathogen, and by the rapid deployment of efficient antiviral defense reactions (incompatible interaction). Late and weak host defense reactions, however, result in susceptibility and systemic spread of the virus (compatible interaction). The investigation of the mechanisms whereby pathogenic viruses elicit defense responses in plant cells is of key importance to the understanding of plant virus resistance. Currently, transcriptional reprogramming in virus-infected plants is generally investigated by transcriptome-wide, next-generation RNA sequencing (RNA-Seq) methods^24–26^. The complete pepper genome has recently been sequenced by three independent research teams providing detailed genetic information of about 35,000 pepper genes arranged on 12 chromosomes^27–29^. These novel pepper genetic databases allow the use of transcriptome-wide, high performance analytical methods like RNA-Seq in virus-infected pepper leaves. This unbiased approach can provide genuinely novel information about defense genes and regulatory mechanisms^26,30^. However, relatively few RNA-Seq studies have been carried out yet with tobamovirus-infected plants^26^. In pepper, comprehensive RNA-Seq transcriptome analyses were carried out following inoculations with different strains of TMV and *Pepper mottle virus* (PepMov, a potyvirus)^31,32^, with PMMoV^33^ as well as with *Capsicum chlorosis virus* (CaCV, a tospovirus)^34^ and *Cucumber mosaic virus* (CMV, a cucumovirus)^35^. No transcriptome-wide analysis has been carried out yet with ObPV.

In the present study, we used an Illumina RNA-Seq method to explore transcriptomic changes in a pepper cultivar harboring the *L*^*3*^ resistance gene following inoculation with two different tobamoviruses. ObPV inoculation led to the appearance of local necrotic lesions (incompatible interaction, HR), while an *L*^*3*^ resistance-breaking strain of PMMoV caused no visible symptoms (compatible interaction)^2,36^. Previously we already used these pepper-tobamovirus interactions to characterize pepper genes related to lipid and oxylipin metabolism^37–39^. Furthermore, photosynthetic and hormonal responses^36,40^ as well as changes in sugar metabolism^41^ were also studied in ObPV- and PMMoV-inoculated pepper leaves. In our present investigations, the comparison of global transcriptomic responses in an incompatible and a compatible pepper-virus interaction revealed novel host genes and regulatory mechanisms of defense pathways that may play substantial roles in the resistance of pepper against tobamoviruses.

## Results and discussion

### Disease symptoms and virus multiplication

ObPV inoculation resulted in the development of typical local necrotic lesions (HR) on the inoculated pepper leaves at 3 days post-inoculation (dpi). These visual symptoms were already shown in an earlier publication^36^. ObPV-inoculated leaves were shed approximately at 7 dpi, but the plants survived the inoculation. In contrast, PMMoV was able to spread into the whole plant, the infection became systemic without any visible disease symptoms according to earlier results. Leaf abscission was not observed at PMMoV inoculated leaves^2,36^.

**Figure 1:**
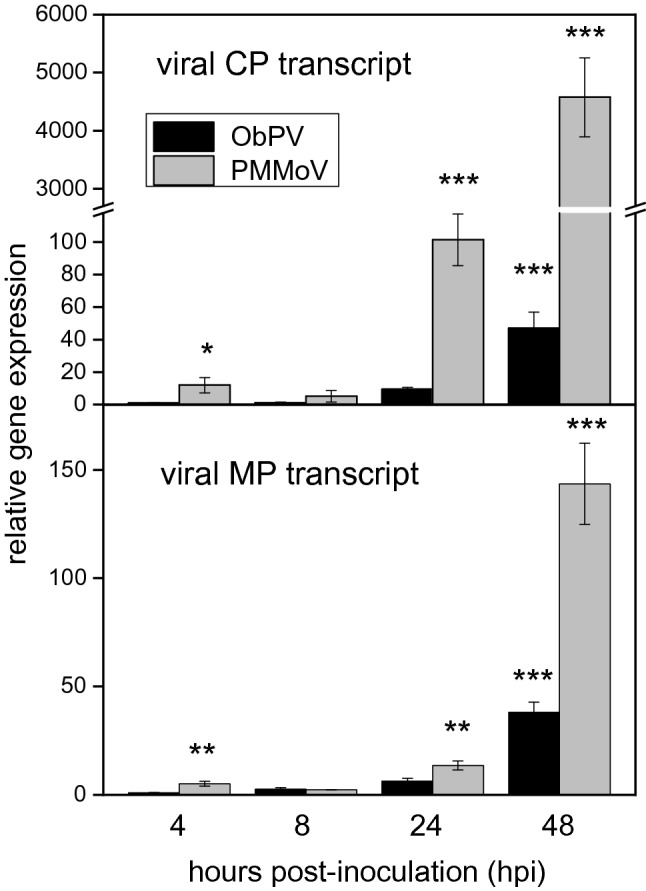
Multiplication of *Obuda pepper virus* (ObPV) and *Pepper mild mottle virus* (PMMoV) in the inoculated pepper leaves at different time points following the inoculations. The amount of mRNAs encoding viral movement proteins (MP) and coat proteins (CP) of ObPV and PMMoV was detected by real-time RT-qPCR (for primers see Supplementary Table S7). Reverse transcriptions (RT) of viral RNAs were carried out by using the reverse primers of the MP or CP specific primer pairs instead of an Oligo(dT) primer. Mean values of three independent experiments are shown ± SD. Relative expression values were calculated by dividing each expression value with the expression of ObPV genes at 4 hpi. The symbols *, ** and *** show significant differences between ObPV- and PMMoV-inoculated plants at *P* < 5%, < 1% and < 0.1%, respectively.

We isolated total RNA extracts from the ObPV-, PMMoV- and mock-inoculated pepper leaves at 4, 8, 24 and 48 h post-inoculation (hpi), so we obtained 12 RNA libraries. First, we investigated the multiplication of ObPV and PMMoV in the inoculated pepper leaves by measuring the amount of mRNAs encoding viral movement proteins (MP) and coat-proteins (CP) in the total leaf RNA extracts by quantitative, real-time RT-PCR. Generally, the accumulation of PMMoV was markedly stronger than that of ObPV in spite of the absence of any symptoms on PMMoV-inoculated leaves. This result confirmed earlier experiments with other methods^2,38^. The transcript abundances of PMMoV *MP* and *CP* genes were 3.8-fold and 97-fold higher than those of ObPV at 48 hpi, respectively (Fig. 1). The expression of ObPV *MP* and *CP* genes gradually increased in the inoculated leaves between 4 and 48 hpi. Interestingly, the amounts of mRNAs encoding MP and CP of PMMoV slightly but significantly declined at 8 hpi as compared to 4 hpi, but later they very significantly rose at 24 and 48 hpi (Fig. 1).

### Identification of differentially expressed genes (DEGs)

**Figure 2:**
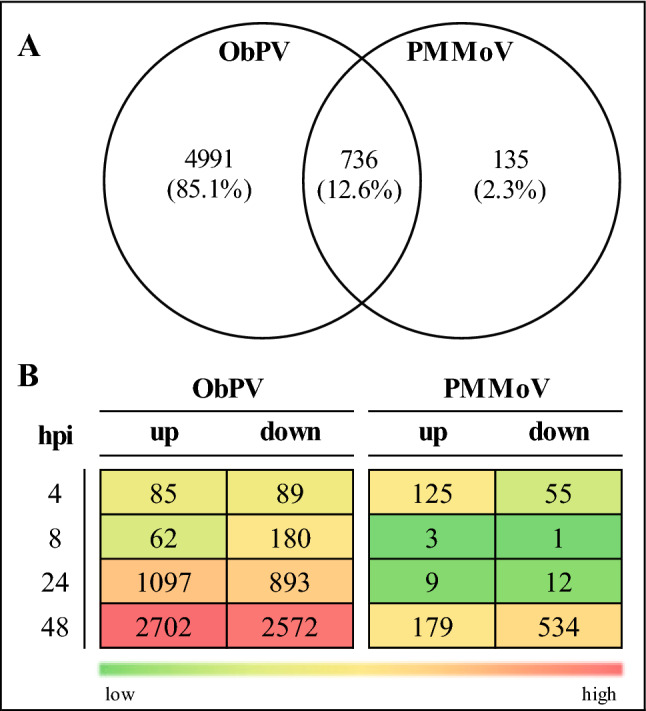
Distribution of differentially expressed genes (DEGs) in pepper leaves following inoculations with *Obuda pepper virus* (ObPV) and *Pepper mild mottle virus* (PMMoV). (**A**) Venn-diagram of all unique and common DEGs in ObPV- and PMMoV-inoculated pepper leaves. (**B**) Number of up- and down-regulated DEGs in ObPV- and PMMoV-inoculated pepper leaves at 4, 8, 24 and 48 h post-inoculation (hpi) as compared to the corresponding mock-inoculated samples (threshold values: *p* ≤ 0.01 and − 1 ≥ log2 fold change (FC) ≥ 1). Color scale corresponds to the number of DEGs.

Gene expression patterns of the 12 RNA libraries were investigated by a transcriptome-wide RNA-Seq method and so we obtained expression data of about 31,000 pepper genes in each RNA library. General mapping statistics are available in Supplementary Table S1. For each RNA library we established a list of differentially expressed genes (DEGs) by filtering all expression data with two threshold values: *p* ≤ 0.01 and − 1 ≥ log2 fold change (FC) ≥ 1. The cumulative list of DEGs for all 12 libraries as well as the DEG lists separately for each time points are presented in Supplementary Table S2. Generally, ObPV (incompatible interaction) exerted a much stronger effect on gene expression patterns than PMMoV (compatible interaction) (Fig. 2). A Venn-diagram shows that the proportion of overlapping DEGs between ObPV- and PMMoV-inoculated leaves was only 12.6% (Fig. 2A). The group of overlapping DEGs showed a high diversity, the most characteristic feature was the down-regulation of a large number of genes related to photosynthesis and carbon fixation. In addition, several overlapping genes were associated with porphyrin and chlorophyll metabolism, hormone signal transduction and ribosomal functions (Supplementary Table S2). In ObPV-inoculated leaves, the number of DEGs generally increased concomitantly with the development of infection, in the case of both up- or down-regulated genes (Fig. 2B). In contrast, the number of DEGs was intriguingly low in PMMoV-inoculated leaves at 8 and 24 hpi but then rapidly increased at 48 hpi, mostly in the case of down-regulated genes (Fig. 2B). An earlier transcriptome-wide analysis of pepper leaves inoculated with the PMMoV-HLD isolate identified 172 up-regulated and 25 suppressed DEGs^33^. However, in this study the RNA samples were taken at 9 days post-PMMoV-inoculation, therefore these results are not comparable to ours.

The first overview of DEG lists showed that large, diverse sets of genes are activated or down-regulated by both ObPV and PMMoV (except for 8 and 24 hpi for PMMoV). The most predominantly up- and down-regulated genes identified in ObPV-inoculated pepper leaves at 24 hpi are shown in Table 1. Upon ObPV inoculation, the most strongly induced pepper genes encoded specific transcription factors, pathogenesis-related (PR) proteins, enzymes of phenylpropanoid and terpenoid biosynthesis, enzymes of ethylene biosynthesis, fatty acid desaturases (FADs) and glutathione S-transferases (GSTs) (Table 1). The massive induction of several pepper *FADs* by ObPV was already analyzed in detail^39^. Intriguingly, almost all strongly ObPV-induced genes displayed also a marked up-regulation by 5 mM ethephon (ethylene precursor) (Table 1; Supplementary Table S2). These results clearly showed the prominent importance of ethylene signaling in the up-regulation of defense genes in ObPV-inoculated leaves. The early and strong accumulation of ethylene in ObPV-inoculated pepper leaves at 24 hpi was already reported^2^. Furthermore, numerous strongly ObPV-inducible pepper genes showed marked induction by salicylic acid (Supplementary Table S2), which confirmed its well-known role in antiviral defense. Methyl-jasmonate and ABA, however, seem to play a minor role in the inducibility of defense genes (Supplementary Table S2). In contrast to ObPV, PMMoV induced genes encoding a polyphenol oxidase, PR-proteins, a smotin-like protein, a branched-chain-amino-acid aminotransferase, an expansin and an aquaporin at 24 hpi (Supplementary Table S2).

**Table 1:** The most predominantly up- and down-regulated DEGs in pepper leaves at 24 h following inoculation with *Obuda pepper virus* (ObPV) as detected by RNA-Seq (*p* ≤ 0.01).

Gene name	NCBI accession number	Function/similarity	Fold-change in log2 scale	
			ObPV inoculation	5 mM ethephon
**Up-regulated genes**				
*CaEAS*	XM_016700945	5-Epiaristolochene synthase-like	10.59	3.66
*CaCXE20*	XM_016713862	Probable carboxylesterase 120	9.57	5.81
*CaFAD*	XM_016696442	Delta(12)-acyl-lipid-desaturase-like	8.87	4.54
*CaUFGT2*	XM_016712585	Anthocyanidin 3-O-glucosyltransferase 2-like	8.57	n.ch
*CaCYP71D7*	XM_016696076	Cytochrome P450 71D7-like	8.38	4.01
*CaGST*	XM_016682087	Probable glutathione S-transferase	8.32	5.21
*CaCCOAOMT*	XM_016705577	Caffeoyl-CoA O-methyltransferase-like	7.93	4.49
*LOC107856987*	XM_016701944	Uncharacterized LOC107856987	7.69	2.45
*CaEAS*	XM_016703676	5-Epiaristolochene synthase-like	7.69	5.21
*CaEP1*	XM_016724042	Epidermis-specific secreted glycoprotein EP1-like	7.68	4.51
*CaPTI*	XM_016706561	Pathogenesis-related transcriptional activator	7.63	2.10
*LOC107877386*	XM_016724039	Uncharacterized LOC107877386	7.61	5.98
*CaEAS2*	XM_016696793	5-Epi-aristolochene synthase 2-like	7.55	n.d
*CaACLA2*	XM_016687133	ATP-citrate synthase alpha chain protein 2-like	7.37	2.44
*LOC107848698*	XM_016693469	Uncharacterized LOC107848698	7.24	1.35
*CaEAS*	XM_016696077	5-Epiaristolochene synthase	7.22	4.39
*CaSTH-2*	XM_016698168	Pathogenesis-related protein STH-2-like	7.18	n.ch
*CaWRKY43*	XM_016706845	Probable WRKY transcription factor 43	7.17	2.36
*CaGST*	XM_016686963	Probable glutathione S-transferase	7.06	5.04
*CaGDS*	XM_016723925	(-)-Germacrene D synthase-like	6.98	2.20
*CaGELP*	XM_016712302	GDSL esterase/lipase At4g10955-like	6.95	4.62
*CaZAT11*	XM_016719311	Zinc finger protein ZAT11-like	6.89	1.87
*CaPRP-DC2.15*	XM_016697601	14 kDa proline-rich protein DC2.15-like	6.73	3.98
*CaCYP82A3*	XM_016713202	Cytochrome P450 82A3-like	6.73	3.68
*CaUSP*	XM_016686895	Uncharacterized protein C167.05-like	6.71	5.17
**Down-regulated genes**				
*CaZOG1*	XM_016692078	Zeatin O-glucosyltransferase-like	− 4.46	n.ch
*CaMT2A*	XM_016720380	Metallothionein-like protein type 2	− 4.50	n.d
*CaPXC3*	XM_016716461	Leucine-rich repeat receptor-like kinase PXC3	− 4.50	1.76
*CaEDR2L*	XM_016722366	ENHANCED DISEASE RESISTANCE 2-like	− 4.51	1.54
*CaRSI-1*	XM_016717019	Protein RSI-1	− 4.54	1.91
*CaEXPA10*	XM_016705906	Expansin-A10	− 4.56	2.46
*LOC107849608*	XM_016694165	Uncharacterized LOC107849608	− 4.59	n.ch
*CaCysPrx*	XM_016693244	1-Cys peroxiredoxin	− 4.65	n.d
*CaUFGT2*	XM_016706149	Anthocyanidin 3-O-glucosyltransferase 2-like	− 4.67	− 4.47
*CaGA3OX1*	XM_016721543	Gibberellin 3-beta-dioxygenase 1-like	− 4.79	n.d
*CaCSLH1*	XM_016725131	Cellulose synthase-like protein H1	− 4.84	n.ch
*CaAGP4*	XM_016716308	Classical arabinogalactan protein 4-like	− 4.87	n.d
*CaLolp11-like*	XM_016719219	Major pollen allergen Lol p 11-like	− 4.91	n.d
*CaCOL16*	XM_016693241	Zinc finger protein CONSTANS-LIKE 16-like	− 4.91	n.ch
*CaAGP18*	XM_016705498	Lysine-rich arabinogalactan protein 18-like	− 4.96	− 1.03
*CaUGT73C3*	XM_016687645	UDP-glycosyltransferase 73C3-like	− 5.24	− 1.93
*CaGATL3*	XM_016692835	Probable galacturonosyltransferase-like 3	− 5.34	− 1.49
*CaXTH32*	XM_016687318	Xyloglucan endotransglucosylase/hydrolase 32	− 5.44	− 1.27
*CaNAS*	XM_016683300	Nicotianamine synthase	− 5.50	− 1.41
*CaEXPA11*	XM_016712858	Expansin-A11-like	− 5.55	− 2.35
*CaRNase-LE*	XM_016693066	Extracellular ribonuclease LE	− 5.63	2.31
*CaGAST1-like*	XM_016719007	Protein GAST1-like	− 5.70	− 3.88
*CaNPF7.3*	XM_016695629	Protein NRT1/ PTR FAMILY 7.3-like	− 6.14	n.d
*CaPRP-DC2.15*	XM_016702234	14 kDa proline-rich protein DC2.15-like	− 6.37	2.02
*CaCHX20*	XM_016687096	Cation/H( +) antiporter 20-like	− 6.75	− 2.93

Gene expression values were compared to mock-inoculated leaves. Expression data of pepper leaves treated with 5 mM ethephon (ethylene precursor) for 6 h were calculated from publicly available raw data in the Sequence Read Archive (SRP265260) published by Lee et al. (2020)^42^. Ethephon-induced gene expression values were compared to water-treated control. Abbreviations: n.ch.: no significant change; n.d.: no data available.

### Functional enrichment analysis of DEGs

**Figure 3:**
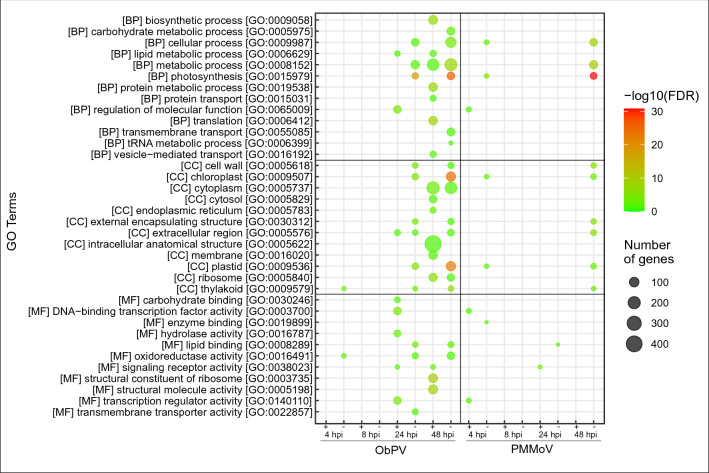
Gene Ontology (GO) classification of differentially expressed genes (DEGs) in pepper leaves inoculated with *Obuda pepper virus* (ObPV) and *Pepper mild mottle virus* (PMMoV) at 4, 8, 24 and 48 h post-inoculation (hpi). The number of DEGs in the various pathways is indicated by the size of colored circles, while the circle colors represent the statistical significance of gene enrichment (False Discovery Rate, FDR).

**Figure 4:**
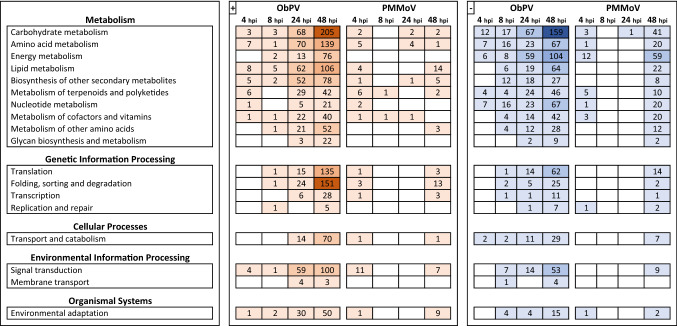
KEGG annotation of differentially expressed genes (DEGs) in pepper leaves inoculated with *Obuda pepper virus* (ObPV) and *Pepper mild mottle virus* (PMMoV) at 4, 8, 24 and 48 h post-inoculation (hpi). Color codes: brown: up-regulated genes; blue: down-regulated genes; empty cells: no genes with significantly changed expression. The KEGG pathway database^43^ was used with the permission of Kanehisa Laboratories, Japan.

We carried out a Gene Ontology (GO) analysis, in which 4498 out of 5862 DEGs were annotated and 36 GO terms were found to be significantly overrepresented. In the ObPV-inoculated samples at 48 hpi the ‘Photosynthesis [GO:0015979], ‘Chloroplast [GO:0009507]’ and ‘Plastid [GO:0009536]’ were the three most significantly overrepresented GO terms (Fig. 3; Supplementary Table S3). In addition, ‘Carbohydrate metabolic process [GO: GO:0005975]’, ‘Lipid metabolic process [GO:0006629]’ and ‘Protein metabolic process [GO:0019538]’ were the most revealing terms, especially during the later stages of infection. These general observations were confirmed by KEGG analysis of pepper metabolic routes^43^. The KEGG annotation classified 1557 pepper genes that were significantly influenced by ObPV or PMMoV in 124 metabolic pathways, which were ordered into 18 pathway classes (Supplementary Table S4). Genes in the ‘Amino acid metabolism’, ‘Carbohydrate metabolism’, ‘Energy metabolism’, ‘Lipid metabolism’, and ‘Biosynthesis of other secondary metabolites’ pathways were generally much stronger influenced by ObPV samples than by PMMoV. In addition, genes in the ‘Translation’ and ‘Folding, sorting and degradation’ pathways were markedly enriched by ObPV at 24 and 48 hpi (Fig. 4; Supplementary Table S4). The detailed KEGG analysis showed that the most prominent effect of ObPV inoculation was the robust suppression of a large number of genes related to photosynthesis, carbon fixation and photorespiration (Supplementary Table S4). Down-regulation of photosynthesis genes have often been observed in virus-infected plants, but the exact mechanisms of this down-regulation are not known^26,44^. In addition, genes related to porphyrin and chlorophyll metabolism, carotenoid biosynthesis and starch biosynthesis were also considerably suppressed by ObPV. Earlier we already observed the strong inhibition of photochemical energy conversion and a declining chlorophyll *a* content in ObPV-inoculated pepper leaves^36,41^. On the other hand, ObPV strongly induced several energy-producing pathways like glycolysis, citrate cycle, oxidative phosphorylation and fatty acid degradation (Supplementary Table S4). During virus infection the leaves have an increased energy demand probably to the biosynthesis of virus-induced defense compounds^44^. We observed also the strong up-regulation of several invertase (beta-fructofuranosidase) genes by ObPV (*CaCWINV1*/XM_016688054; *CaAIV-18*/XM_016709343; *CaBRFUCT*/XM_016713500; *CaINVA*/XM_016717981) (Supplementary Table S2), which also indicated the elevated energy demand of virus-inoculated leaves^45^. In accordance with the induction of invertase genes, we demonstrated earlier the marked accumulation of glucose and fructose in ObPV-inoculated leaves^41^. Furthermore, ObPV markedly induced the MAP-kinase signaling cascades, calcium-dependent signaling, the phosphatidylinositol signaling system, protein catabolism, protein processing in the endoplasmic reticulum, cysteine and methionine metabolism, tyrosine and phenylalanine metabolism, phenylpropanoid biosynthesis, glycerolipid metabolism, and terpenoid biosynthesis (Supplementary Table S4). In contrast to ObPV, PMMoV exerted much weaker effects on pepper metabolic routes. PMMoV also suppressed genes associated with photosynthesis, carbon fixation, photorespiration as well as porphyrin and chlorophyll metabolism but to a much lesser extent than ObPV. PMMoV inoculation induced Ca-dependent signaling, protein processing in the endoplasmic reticulum, and glycerolipid metabolism. However, PMMoV did not influence significantly the genes related to energy production (cellular respiration and beta oxidation), MAP-kinase signaling and the biosynthesis of defense compounds like phenylpropanoids (Supplementary Table S4).

### Detailed analysis of six selected DEG groups

For a further, detailed analysis we arbitrarily selected six groups of pepper genes that were ordered into separate lists by manual curation. These datasets comprise genes encoding immune receptors, transcription factors, pathogenesis-related proteins (PR-proteins), as well as enzymes of terpenoid biosynthesis, ethylene metabolism and sulfur metabolism (Supplementary Table S5). We suppose that these robustly virus-inducible genes encode key factors of antiviral defense.

1. Immune receptors

Resistance (R)-proteins generally contain a nucleotide-binding (NB) motif and a leucine-rich repeat (LRR) motif. In pepper, 755 genes were identified that encoded potential NB-LRR-coding protein sequences^46^. The *L*^*3*^ gene (GenBank LOC107854789)^12^ that confers resistance to tobamoviruses was identified among the genes detected in our RNA-Seq experiments, but the expression of *L*^*3*^ gene was not altered by ObPV or PMMoV (data not shown). Nevertheless, we found two close *L*^*3*^ homologs, which were significantly up-regulated by ObPV at 48 hpi (*CaRPP13L1*/XM_016716577, *CaRPP13L3*/XM_016724966), Supplementary Table S5). The R-proteins encoded by these genes have a typical CC-NBS-LRR structure^12^. In addition, we found two resistance genes encoding TIR-NBS-LRR type R-proteins (TMV resistance protein *N*-like genes), which were also markedly activated by ObPV at 24 and 48 hpi (*CaN_XI*/XM_016696219 and *CaN*/XM_016696926, Supplementary Table S5). PMMoV did not influence the expression of these *R*-genes. The exact function of these proteins in tobamovirus-inoculated pepper leaves is unknown yet.

Beside the above R-proteins, which are mainly located in the cytoplasm, numerous cell surface receptor kinases play critical roles in virus resistance as immune receptors^47,48^. We identified numerous ObPV-induced genes that encode leucine-rich repeat receptor protein kinases, serine/threonine-protein receptor kinases, lectin receptor kinases (LecRKs) and other types of receptor kinases (Supplementary Table S5). These receptor kinases are typically transmembrane proteins, which can perceive diverse extracellular signals and transduce them by an intracellular protein kinase domain towards the nucleus by various signal transduction pathways. It is not known whether these plasma membrane-anchored receptors sense viral components through their extracellular or intracellular domain^47^. A particularly large set of *LecRKs* genes were robustly induced by ObPV. LecRKs possess an extracellular domain sharing sequence similarity with legume lectins. This lectin-like domain can bind various molecules like oligosaccharides or plant hormones^49^. Interestingly, suppressed expression of the *LecRK-S.5* gene in pepper led to enhanced susceptibility against TMV and PMMoV^50^.

(2) Transcription factors

Generally, six transcription factor families are associated with plant defense mechanisms: AP2/ERF, bHLH, bZIP, MYB, NAC and WRKY^51^. The pepper genome contains 2,153 transcription factors (6.25% of the total genes), which were classified into 80 gene families^27^. In our studies, genes encoding the AP2/ERF, heat-shock transcription factor, NAC, WRKY and ZAT families were robustly up-regulated by ObPV, whereas those of the bHLH and TCP families were suppressed. PMMoV exerted only a negligible effect on the expression of transcription factor genes (Supplementary Table S5).

In the pepper genome 142 ethylene-responsible transcription factors (ERFs) were identified^52^. In *A. thaliana*, ERFs were classified into 12 groups. Pathogen-inducible *A. thaliana ERF* genes typically belong to group IX^53^. In our work, several strongly ObPV-inducible pepper *ERFs* (*CaERF1A*/XM_016714653, *CaPTI5*/XM_016706561, *CaERF1B*/XM_016717675) showed homology to group IX genes of *A. thaliana*. Interestingly, constitutive overexpression of the tobacco *ERF5* gene (homolog to pepper gene *CaERF1B*/XM_016717675) led to enhanced resistance to TMV, demonstrated by strongly reduced viral RNA level^54^.

Twenty-five heat shock transcription factors (HSTFs) were identified in pepper^55^. ObPV significantly activated the expression of ten *HSTFs*, particularly those belonging to the B class of *HSTFs*. Thus, the genes *CaHSFB3*/XM_016688344, *CaHSFB3/*XM_016716424 and *CaHSFB1*/XM_016705606 were up-regulated 23-, 13- and 7.5-fold by ObPV at 24 hpi, respectively (Supplementary Table S5). Although heat shock transcription factors are known to have a pivotal role in the regulation of immune response^56^, very limited information is available about their functions in virus-infected plants^57,58^.

The WRKY family is well characterized in pepper^59^, and their participation in pepper-tobamovirus interactions is also amply demonstrated^5,60^. In our studies, 23 *WRKY* genes were activated by ObPV at 24 and 48 hpi, and some of them were among the most strongly ObPV-activated transcription factor genes. In contrast, ObPV suppressed the expression of only three *WRKYs* (in two cases transiently). PMMoV slightly induced five *WRKY* genes (Supplementary Table S5). Furthermore, 13 *NAC* and 8 *ZAT* genes were up-regulated by ObPV, particularly robustly in the case of *ZATs*. NAC and ZAT transcription factors are known to participate in antiviral defense^23,61^, but few information is available about them in pepper. The above results warrant further studies on the role of ObPV-induced transcription factors in the tobamovirus resistance of pepper.

(3) Pathogenesis-related (PR) proteins

The large and diverse group of PR-proteins were classified into 17 families^62^. In our studies, numerous genes encoding PR-1 proteins, chitinases (PR-3), endochitinases (PR-4), osmotins (PR-5), proteinase inhibitors (PR-6), lignin-forming peroxidases (PR-9), and ribonuclease-like proteins (PR-10) were markedly induced upon ObPV inoculation at 24 and 48 hpi, whereas those encoding glucanases (PR-2), aspartic endoproteases (PR-7), defensins (PR-12) and lipid transfer proteins (PR-14) were mostly suppressed. In contrast to ObPV, PMMoV inoculation generally exerted a much weaker effect on *PR* genes, however it suppressed several defensin-coding *PR-12* genes (Supplementary Table S5). In an earlier study, the expression of the pepper *CaPR-4*, *CaPR-6* (*CaPIN-II*), *CaPR-7* and *CaPR-10* genes was markedly induced during an incompatible pepper-TMV interaction^63^. In *Capsicum chinense L*^*3*^-PMMoV interactions CcPR-1, CcPR-2, CcPR-3, CcPR-5, CcPR-9, germin-like protein (CcPR-16), and CcPRp27 (CcPR-17) were identified in a proteomic analysis. Three PR protein isoforms (CcPR-2, CcPR-5 and a basic CcPR-1) were specifically induced during an incompatible *C. chinense*-PMMoV interaction^16^.

In our studies, the *PR-3*, *PR-9* and *PR-10* gene families were particularly highly induced by ObPV (Supplementary Table S5). Notably, seven *PR-10* genes were induced by ObPV. The most strongly induced *CaPR-10* gene (XM_016710983) was activated 79- and 596-fold by ObPV at 24 and 48 hpi, respectively, as compared to control values. The RNA-Seq analysis of CaCV-infected pepper leaves revealed the high CaCV-inducibility of *PR-3*, *PR-9* and *PR-10* genes^34^. In an incompatible pepper-TMV interaction PR-10 transcripts were also robustly induced. The recombinant PR-10 protein exhibited a ribonuclease activity against TMV RNA. In addition, TMV inoculation led to the phosphorylation of PR-10, which increased its ribonucleolytic activity^64^. We assume that PR-10 proteins may participate in the defense of pepper against tobamoviruses due to their ribonuclease activity.

(4) Terpenoid biosynthesis

Upon ObPV inoculation, a large number of genes participating in the biosynthesis of various terpenoids (also called isoprenoids) were among the most robustly activated in pepper leaves. The carbon backbone of terpenoids is synthesized by the mevalonate pathway, which produces isopentenyl 5-diphosphate (IPP) from acetyl-CoA, through mevalonic acid. The three steps of the upper mevalonate pathway are catalyzed by acetyl-CoA acetyltransferase (also known as acetoacetyl-CoA thiolase), hydroxymethylglutaryl-CoA synthase (HMGCS) and 3-hydroxy-3-methylglutaryl-coenzyme A reductase (HMGCR) enzymes, finally resulting mevalonic acid^65–67^. In our RNA-Seq studies the expression of an acetyl-CoA acetyltransferase gene and two *HMGCS* genes were strongly induced by ObPV at 24 and 48 hpi (Supplementary Table S5). Furthermore, we detected the expression of 5 *HMGCR* genes in pepper leaves, from which four *HMGCRs* were markedly upregulated by ObPV. The expression of a *HMGCR* gene (*CaHMG-CoA*/XM_016702518) was 60- and 326-fold higher in ObPV-inoculated leaves at 24 and 48 hpi, respectively, than in control leaves. The expression of these was not influenced significantly by PMMoV inoculation.

In the further steps of IPP biosynthesis, mevalonic acid is phosphorylated and decarboxylated to generate IPP by mevalonate kinase, mevalonate 5-phosphate kinase, and mevalonate 5-diphosphate decarboxylase^65^. IPP can be converted to its isomer dimethylallyl diphosphate (DMAPP) by IPP isomerases. Upon ObPV-inoculation, the coding genes of the four above-mentioned enzymes were also markedly upregulated (Supplementary Table S5). During terpenoid biosynthesis, three IPPs are condensed sequentially to DMAPP to produce geranyl diphosphate (GPP, C_10_), farnesyl diphosphate (FPP, C_15_) and geranylgeranyl diphosphate (GGPP, C_20_)^68^. ObPV inoculation strongly induced two farnesyl pyrophosphate synthase genes in pepper leaves already at 1 dpi (38- and 26-fold in the case of *CaFPS1*/XM_016689996 and *CaFPS1/*XM_016709849, respectively). Intriguingly, while three chloroplastic geranylgeranyl pyrophosphate synthase (*GGPPS*) genes were markedly down-regulated after ObPV inoculation, one *GGPPS* gene (*CaGGR*/XM_016701317) was activated as soon as 4 hpi by ObPV.

In infected plants, FPP is utilized to the biosynthesis of sesquiterpene (C_15_) phytoalexins. First, 5-epiaristolochene synthase (EAS) enzymes catalyze the cyclization of FPP to 5-epi-aristolochene^69–71^. In our work, five *EAS* genes were massively up-regulated following ObPV inoculation and some of them were among the most robustly ObPV-inducible genes (up to 2074-fold induction at 48 hpi, *CaEAS2*/NM_001324691). The transcript abundance of other sesquiterpene cyclases like alpha-farnesene synthase, germacrene synthase and vetispiradiene synthase genes also substantially increased by ObPV (Supplementary Table S5). In the following reaction, 5-epi-aristolochene is converted to capsidiol (C_15_ phytoalexin) by 5-epi-aristolochene-1,3-dihydroxylase (EAH) enzymes^70,72^. Interestingly, cytochrome P450 71D (CYP71D) enzymes were shown to possess EAH activity in tobacco^72^. We detected the transcripts of five *CYP71D7* genes in the pepper transcriptome and all of them were substantially activated by ObPV, up to a 2296-fold induction in the case of a *CYP71D7* gene (*CaCYP71D7*/XM_016694256) at 48 hpi. Furthermore, two premnaspirodiene oxygenase genes were also strongly induced by ObPV. Premnaspirodiene oxygenases are also cytochrome P450 enzymes that catalyze the hydroxylations of diverse sesquiterpenes^73^.

We hypothesize that upon ObPV inoculation a blend of several sesquiterpene phytoalexins accumulates in pepper leaves. The strong increase of capsidiol content was already observed a long time ago in pepper and tobacco leaves following elicitor treatments and microbial infections^70,74–76^. Capsidiol and other sesquiterpenes showed a moderate antiviral effect in tobacco against TMV^77^. Furthermore, capsidiol 3-acetate also possess antiviral activity against *Potato Virus X* (PVX) in *Nicotiana benthamiana*^76^. Interestingly, silencing of an ethylene-responsive transcription factor gene (*NaERF2*) strongly reduced the expression of *EAS* and *EAH* genes as well as the capsidiol content in *Nicotiana attenuata*^78^. The closest pepper homolog of *NaERF2* was identified in our studies as a strongly ObPV-inducible *ERF* gene (*CaERF1A*/XM_016714653), Supplementary Table S5). These results show that capsidiol biosynthesis may be transcriptionally regulated by an ERF transcription factor in ObPV-inoculated pepper leaves.

Intriguingly, we identified also two beta-amyrin 28-oxidase genes, which were massively induced by ObPV at 24 and 48 hpi. These genes are identical to cytochrome *P450 716A* genes, which participate in the biosynthesis of various triterpenoids (C_30_ compounds) like oleanolic acid, ursolic acid and betulinic acid^79^. In addition, we found a strongly ObPV-inducible beta-amyrin 11-oxidase gene (cytochrome P450 88D-like), which is involved in the biosynthesis of glycyrrhizin^80^. These triterpenoids possess a broad antiviral spectrum^81^.

(5) Ethylene metabolism

A sharp increase of ethylene production has been often observed in virus infected plants^82^. A rapid and massive accumulation of ethylene was also detected in ObPV-inoculated pepper leaves already at 24 hpi, while the ethylene production was not induced by PMMoV^2^. Apart from ethylene, several other defense hormones were also shown to accumulate in ObPV-inoculated leaves including salicylic acid and jasmonic acid^40^. Ethylene is perceived by specific receptors in the endoplasmic reticulum (ER) and triggers downstream responses^22,83^. Ethylene signaling is initiated by the activation of ethylene biosynthesis. The three-step biosynthetic pathway starts from methionine, which is converted to S-adenosyl-L-methionine (SAM) by S-adenosyl-L-methionine synthase (SAMS) enzymes^84,85^. The following steps are catalyzed by 1-aminocyclopropane-1-carboxylate synthase (ACS) and 1-aminocyclopropane-1-carboxylate oxidase (ACO) enzymes^86^. In our present studies, genes encoding a homocysteine S-methyltransferase (*CaHMT-2*/XM_016710426) and a SAM synthase (*CaSAM*/XM_016687895) were markedly induced by ObPV (Supplementary Table S5). In addition, two *ACS* genes (*CaACS1*/XM_016710904 and *CaACS2*/XM_016682648) and eleven *ACO* genes were also up-regulated by ObPV (some of them robustly), but not by PMMoV. The early up-regulation of several *ACS* and *ACO* genes at 24 hpi coincides with the marked accumulation of ethylene^2^, so we presume that transcriptional activation of these genes contributes to an increased ethylene production. Interestingly, the *A. thaliana* homologs of the two ObPV-inducible *ACS* genes, which are designated as *AtACS2* and *AtACS6*, were already shown to be specifically pathogen-inducible^84,87^. TMV inoculation also up-regulated the expression of *ACO* and *ACS* genes in resistant tobacco leaves^82,86^.

Several genes of the complex ethylene signaling pathway^88^ were also significantly induced by ObPV. Thus, the transcript abundance of genes encoding three ethylene receptors, the copper transporter RAN1, the protein kinase CTR1 as well as genes encoding two EIN3 proteins and two EIN3-binding F-box proteins were significantly elevated by ObPV. The master regulator EIN3 protein is the last component of the ethylene signal transduction pathway. EIN3 is located in the nucleus where it can regulate the expression of a large number of genes encoding ERFs^83,89^.

(6) Sulfur metabolism

The homeostasis of cysteine, which is the central metabolite of plant sulfur metabolism, plays an essential role in plant immunity^90^. Cysteine serves as precursor of a wide variety of antimicrobial or antioxidative thiol compounds such as defensins, glucosinolates, glutathione (GSH), GSTs, phytoalexins, S-containing volatiles and thionins^91^. The cysteine-containing tripeptide GSH is a major antioxidant in plants. GSH participates in plant defense mechanism not only as antioxidant, but also as a signaling compound^92^. Nevertheless, very few information is available about the impact of virus infections on cysteine or GSH biosynthetic pathways^93,94^. In our studies, KEGG analysis revealed that ObPV inoculation significantly activated the cysteine and GSH biosynthetic pathways (Supplementary Table S4). In the cysteine biosynthesis route, genes encoding an 5'-adenylylsulfate reductase, two serine-O-acetyltransferases and two O-acetylserine-(thiol)-lyases (OAS-TL) were activated by ObPV. Particularly a cysteine synthase gene (*CaCY*S/XM_016682985) was induced, the expression of which increased 11- and 16-fold by ObPV at 24 and 48 hpi, respectively (Supplementary Table S5). Furthermore, the expression of two genes participating in GSH biosynthesis was also activated. PMMoV did not influence significantly the cysteine and GSH pathways.

**Figure 5:**
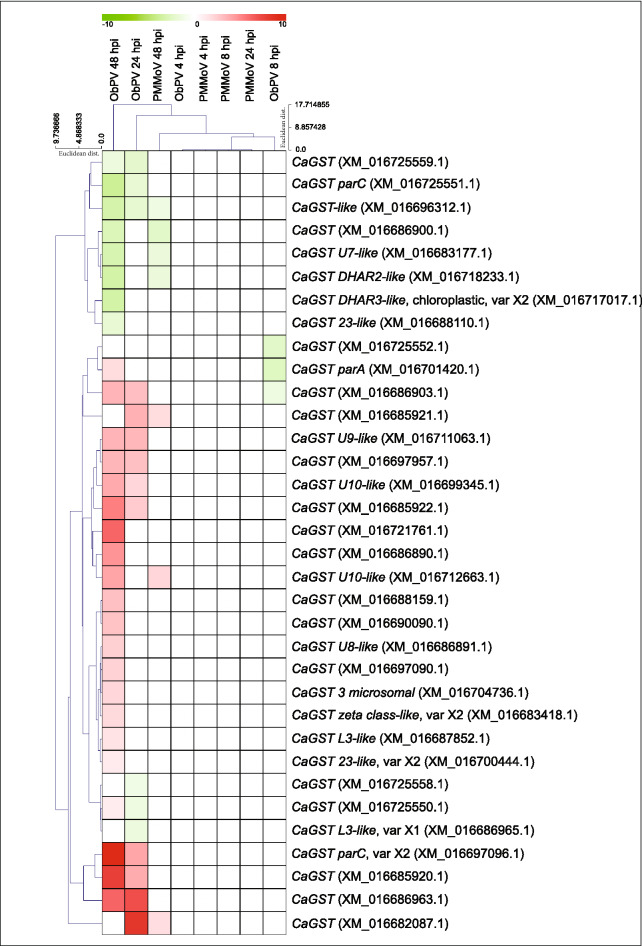
Heat-map representing changes in the expression of 34 glutathione S-transferase (*GST*) genes in pepper leaves inoculated with *Obuda pepper virus* (ObPV) and *Pepper mild mottle virus* (PMMoV) at 4, 8, 24 and 48 h post-inoculation (hpi). Euclidean distance based hierarchical clustering was performed with average-linkage both among each of the *GSTs* and samples.

Notably, *GSTs* were particularly massively induced by ObPV among GSH-related genes. GSTs constitute a large family of soluble proteins that catalyze the covalent binding of GSH to substrates containing a reactive electrophilic centre to form less toxic and more water-soluble conjugates. Although GSTs have a variety of functions, their most likely roles in pathogen-infected plants are the suppression of necrosis by antioxidative reactions, participation in hormone transport and interaction with salicylic acid metabolism^95^. In pepper, eighty-five *GST* genes were identified^96^. In our studies, 22 *GSTs* were highly induced while eleven *GSTs* were suppressed by ObPV, which is represented by a heat-map (Fig. 5). Particularly the genes *CaGST*/XM_016697096 and *CaGST*/XM_016685920 were massively up-regulated by ObPV at 48 hpi, their expressions were 597- and 214-fold higher than in mock samples (Supplementary Table S5). In contrast to *GSTs*, the genes encoding glutathione peroxidases, glutaredoxins, sulfotransferases, thioredoxins and peroxiredoxins were (with some exceptions) markedly suppressed by ObPV.

### Validation of RNA-Seq results by real-time RT-qPCR

**Figure 6:**
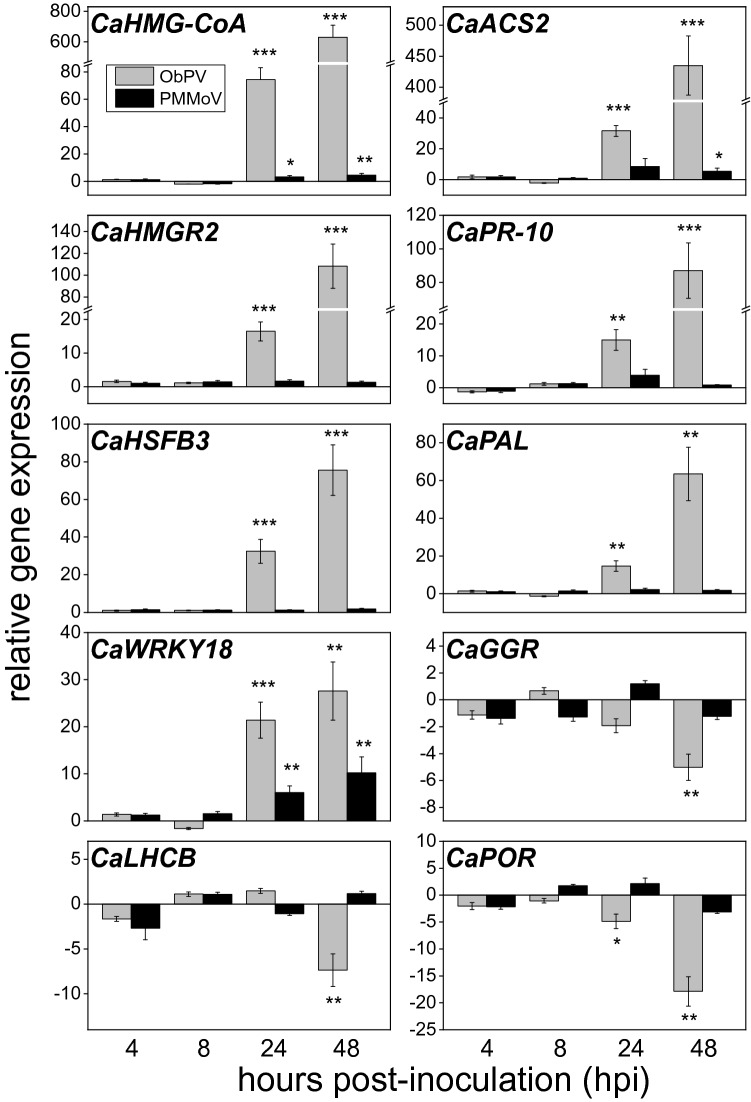
Changes in the relative expression of ten selected pepper genes in ObPV- and PMMoV-inoculated leaves analyzed by real-time RT-qPCR. The ten selected genes and their GenBank accession numbers were the following: hydroxymethylglutaryl-CoA synthase (*CaHMG-CoA/*XM_016702295), 1-aminocyclopropane-1-carboxylate synthase (*CaACS2/*XM_016682648), 3-hydroxy-3-methylglutaryl-coenzyme A reductase (*CaHMGR2/*XM_016702348), pathogenesis-related protein 10 (*CaPR-10*/XM_016710983), heat shock transcription factor (*CaHSFB3/*XM_016688343), phenylalanine ammonia-lyase (*CaPAL*/NM_001324603), WRKY transcription factor 18-like (Ca*WRKY18*/XM_016726661), geranylgeranyl diphosphate reductase (*CaGGR*/XM_016707759), chlorophyll a-b binding protein 1B (*CaLHCB/*XM_016700776) and protochlorophyllide reductase (*CaPOR/*XM_016689752). Expression values of these genes were normalized to those of the reference gene ubiquitin-conjugating enzyme 3 (*CaUBC3*, XM_016720449) and then related to mock-inoculated leaf samples at each time point according to the method of Livak and Schmittgen (2001)^97^. Gray and black columns represent ObPV and PMMoV inoculated leaves, respectively. Mean values of three independent experiments are shown ± SD. The symbols *, ** and *** show significant differences between mock and virus-inoculated plants at *P* < 5%, < 1% and < 0.1%, respectively.

The expression of ten selected genes was analyzed by quantitative, real time RT-PCR (qPCR) in ObPV-, PMMoV- and mock-inoculated leaves at 4, 8, 24 and 48 hpi in order to validate the results of RNA-Seq analyses. Both up- and down-regulated genes were selected to qPCR analyses in order to represent a wide scale of expressional changes during the validation. For qPCR analyses we used the same total RNA preparations that were utilized also for RNA-Seq library preparation. The genes encoding a hydroxymethylglutaryl-CoA synthase (*CaHMG-CoA*/XM_016702295), a 1-aminocyclopropane-1-carboxylate synthase (*CaACS2/*XM_016682648), a 3-hydroxy-3-methylglutaryl-coenzyme A reductase (*CaHMGR2*/XM_016702348), a pathogenesis-related protein 10 (*CaPR-10*/XM_016710983), a heat shock transcription factor (*CaHSFB3*/XM_016688343), a phenylalanine ammonia-lyase (*CaPAL/*NM_001324603), and a WRKY transcription factor (*CaWRKY18*/XM_016726661) were significantly activated by ObPV, whereas genes encoding a geranylgeranyl diphosphate reductase (*CaGGR*/XM_016707759), a chlorophyll a-b binding protein 1B (*CaLHCB*/XM_016700776) and a protochlorophyllide reductase (*CaPOR*/XM_016689752) were markedly suppressed by ObPV (Fig. 6). In contrast to ObPV, PMMoV exerted only a negligible effect on the expression of these genes (Fig. 6).

**Figure 7:**
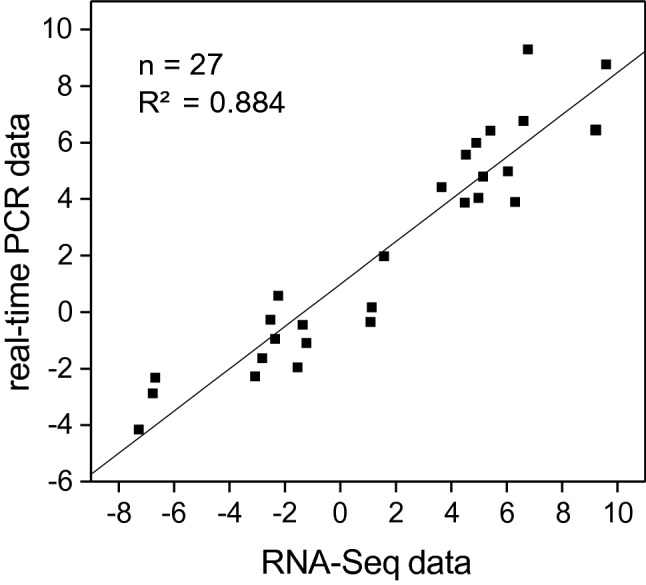
Validation of Illumina RNA-Seq results by real-time RT-qPCR assays for 10 pepper genes. Linear regression coefficient was calculated between gene expression levels obtained from RNA-Seq and RT-qPCR. Fold-changes in the expression of genes are shown on log2 scale at both axes.

Next, we compared to gene expression changes obtained for the ten selected genes by RNA-Seq and qPCR. The fold-changes of gene expression shown on log2 scales presented similar trends in qPCR and RNA-Seq (Fig. 7). A high linear regression coefficient (R^2^ = 0.884) was obtained between the fold-change values obtained by RNA-Seq and qPCR, which proved that the RNA-Seq data were reliable.

### Protein–protein interaction network

**Figure 8:**
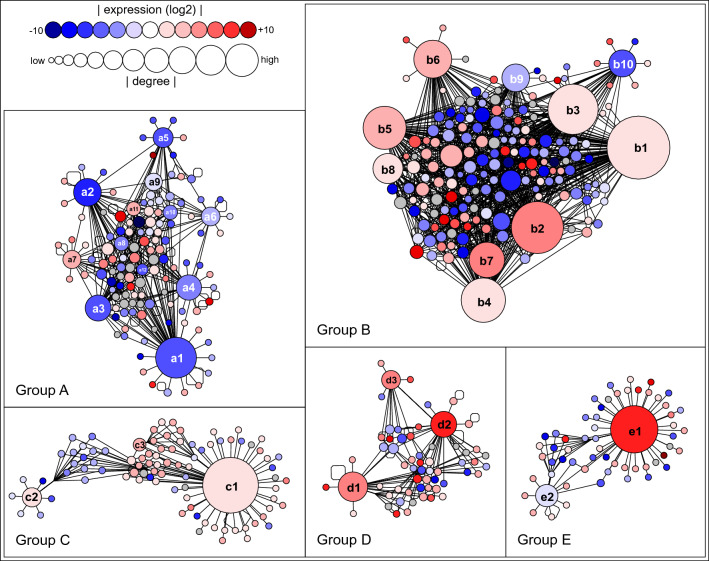
Predicted interactions between pepper proteins. Arabidopsis entries of the BioGRID^98^ protein–protein interaction database were adapted to pepper proteins based on their strong homology. The five separate group of graphs contain 507 proteins (nodes) with 1364 interactions (edges). Nodes with at least ten connections are labelled, namely (**Group A)**; (**a1)** CaTCP14 /XP_016578353, (**a2)** CaTCP15/XP_016546616, (**a3)** CaTCP4/XP_016541752, (**a4)** CaTPR1/XP_016562946, (**a5)** CaGAI/XP_016572500, (**a6)** CaBRZ1/XP_016568620, (**a7)** CaGIF1/XP_016572280, (**a8)** CaTCP21/XP_016564270, (**a9)** CaGAI1/XP_016562448, (**a10)** CaTCP8/XP_016539042, (**a11)** CaRLT1/XP_016550332, (**a12)** CaRBCMT/ XP_016548471. (**Group B)**; (**b1)** CaUBC34/XP_016562414, (**b2)** CaCYP21-4/XP_016542812, (**b3)** CaEMC6/XP_016581021, (**b4)** CaTMEM147/XP_016551565, (**b5)** CaHHP4/XP_016560932, (**b6)** CaCHMP5/XP_016552071, (**b7)** CaUBC32/XP_016551353, (**b8)** CaSPCS-1/XP_016570612, (**b9)** CaACBP/XP_016557647, (**b10)** LOC107865084/XP_016566924. (**Group C); (c1)** CaRPT2A/XP_016542116, (**c2)** CaNUP1/XP_016573762, (**c3)** CaRPN1A/XP_016544974**. (Group D)**; (**d1)** CaMAPK4-2/XP_016538117, (**d2)** CaMPK3/XP_016575773, (**d3)** CaMPK7/XP_016560684. (**Group E)**; (**e1)** CaNAC040/XP_016577960, (**e2)** LOC107876111/XP_016578602. All interactions are shown in Supplementary Table S6. The source code (graphml) of the graphs are deposited to GitHub Gist and accessible online via yEd Live (https://bit.ly/3lxFetd).

Although direct information about the interactions of pepper proteins is not available yet, interactions can be predicted based on the Arabidopsis model. By using the BioGRID^98^ protein–protein interaction database, we determined the possible interactions of 1307 pepper proteins with 2655 connections. Most likely, the expression levels of these genes and the number of their interacting partners are proportional to their importance. Thus, from the complex graph of all predicted pepper protein–protein interactions we cut out 5 subgraphs (Groups A to E) based on centrality, measuring the most linked proteins and their primary neighbors (Fig. 8; Supplementary Table S6). These subgraphs still contain one third of the predicted linkages (507 nodes and 1364 edges). The source code (graphml) of these subgraphs are deposited to GitHub Gist and accessible online via yEd Live (https://bit.ly/3lxFetd). In Group A, we identified five down-regulated TCP transcription factors (Fig. 8, Supplementary Table S6). TCP proteins exert a regulatory role in shaping plant morphology but several recent studies have demonstrated that TCPs also function as a cellular hub in plant defense signaling^99^. Downregulation of CIN-TCPs results in delayed maturation of vegetative organs, which increases the survival of the biotrophic phytoplasma^99^. Interestingly, *miR319* was shown to regulate TCPs transcription factors^100,101^. Furthermore, *Rice ragged stunt virus* (RRSV) up-regulated the expression of *miR319* gene in rice, which led to the suppression of *OsTCP21*. Overexpression of *OsTCP21* increased the resistance of rice to RRSV^102^. We suppose that the suppression of pepper TCP genes was also induced by increased miRNAs levels following ObPV inoculation.

Examining the Group B and C subgraphs, we found that 50S ribosomal proteins, proteasome subunits, parts of the ubiquitin–proteasome system and many LRR receptor-like serine/threonine-protein kinase (LRR-RLKs) proteins were enriched (Fig. 8; Supplementary Table S6). Intriguingly, nine of the ten downregulated LRR-RLK proteins interact with upregulated ubiquitin-conjugating enzymes which may be associated with their rapid degradation. In many cases, ubiquitin-ligases (E3) are phosphorylated by LRR-RLKs. Presumably phosphorylation of ubiquitin-ligases regulates their activity or their interaction with target proteins^103^ but this control may be less effective at low LRR-RLKs level. E3 ligases play important roles in multiple environmental stresses as regulators of salicylic acid, jasmonic acid, and ethylene signaling pathways^104^. Mutations in the Arabidopsis 26S proteasome regulatory subunit coding genes *AtRPN1a* and *AtRPT2A* suppressed *edr2*-associated powdery mildew resistance phenotype^105^. In our study, we found that pepper homologs of these two genes (*CaRPN1a*/XM_016689488 and *CaRPT2A/* XM_016686630) and the expression of twenty other proteasome subunits were up-regulated following ObPV-inoculation, which suggests their important role in plant innate immunity.

In the D subgraph the three proteins having the most connections are mitogen-activated protein kinases (MAPKs) (Fig. 8; Supplementary Table S6). Virus-induced gene silencing (VIGS) of *CaMAPK7* (XM_016705198) significantly enhanced the susceptibility of pepper plants to infection by *Ralstonia solanacearum*^106^. In our work, the expression of this *MAPK* and two other *MAPKs*, *CaMPK3* (XM_016720287) and *CaMPK4* (XM_016682631) were markedly up-regulated by ObPV (Supplementary Table S2). The Arabidopsis homologs of these pepper MAPKs, *AtMPK3* and *AtMPK4* are known to play vital roles in immune responses^107–109^. Previous studies have already reported the crosstalk between MAPKs and TCPs^110^. We presume that MAPKs and ubiquitination^111^ as well as LRR-RLKs and ubiquitination^112^ interact during the regulation of defense reactions in ObPV-inoculated pepper plants.

### Conclusions

We investigated early (4–48 hpi) reactions of pepper plants to inoculations with two different tobamoviruses. Transcriptome profiles of virus-infected leaves were examined by Illumina RNA-Seq method. In the incompatible pepper-ObPV interaction, the strong suppression of photosynthesis-related genes and the massive induction of energy-producing pathways (cellular respiration and beta-oxidation of fatty acids) as well as diverse groups of defense-related genes showed that ObPV inoculation caused a metabolic switch from normal metabolism to a defense mode in the infected pepper leaves. Genes encoding immune receptors, specific transcription factors, PR-proteins, fatty acid desaturases, GSTs, enzymes of ethylene metabolism and terpenoid biosynthesis were particularly strongly up-regulated by ObPV. Intriguingly, PMMoV inoculation (compatible interaction) induced only negligible defense reactions in spite of its rapid replication in the infected leaves. The transcriptome-wide RNA-Seq analysis uncovered a large number of novel, interesting candidate genes for further research. In future work, elucidating the function of newly discovered defense genes will be necessary by both molecular and biochemical methods.

## Materials and methods

### Pepper variety and virus inoculations

The pepper (*Capsicum annuum* L.) cultivar TL 1791 harboring the *L*^*3*^ resistance gene was used for all experiments. Pepper plants were grown under normal greenhouse conditions (18–23 °C; 16 h daylight with 160 µmol m^−2^ s^−1^ supplemental light for 8 h per day; 75–80% relative humidity) and 2-month-old plants were used for the experiments. ObPV and PMMoV rub-inoculations as well as mock-treatments were carried out as described earlier^36^. Three mature middle leaves of each plant were inoculated by ObPV or PMMoV. The ObPV strain was isolated in Hungary (formerly used synonym: Ob strain of Tomato mosaic virus), whereas the *L*^*3*^-resistance-breaking strain of PMMoV was isolated in Louisiana, USA (formerly used synonym: Samsun latent strain of Tobacco mosaic virus)^2,36^. Virus-inoculated and mock-treated plants were kept at 22 °C in a growth chamber with 16/8 h light/dark cycles. Leaf samples were taken from ObPV- and PMMoV-inoculated as well as from mock-inoculated leaves at 4, 8, 24 and 48 h post-inoculation (hpi) for total RNA extraction. At all time points and for all biological replicates, samples were taken from the virus- or mock-inoculated third and fourth true leaves of three plants. Samples were pooled for each treatment, frozen in liquid nitrogen and stored at −70 °C until use. All methods including plants complied with institutional, national and international guidelines and legislation.

### RNA-Seq analyses

Total RNA was extracted from pepper leaves by using the RNeasy Plant Mini Kit (Qiagen, Hilden, Germany). RNA-Seq analysis of RNA samples taken from ObPV-, PMMoV- and mock-inoculated leaves at 4, 8, 24 and 48 hpi were carried out in two independent parallel experiments, so altogether 24 RNA libraries were obtained. The RNA-Seq analyses were carried out by the Xenovea Ltd. (Szeged, Hungary) in collaboration with the Genomics and Bioinformatics Core Facility of the University of Pécs, Hungary. The RNA integrity numbers and RNA concentrations were determined by RNA ScreenTape system with 2200 Tapestation (Agilent Technologies, Santa Clara, CA, USA) and RNA HS Assay Kit with Qubit 3.0 Fluorometer (Thermo Fisher Scientific, Waltham, MA, USA), respectively. For mRNA library construction, QuantSeq 3′ mRNA-Seq Library Prep Kit (FWD) for Illumina (Lexogen, Wien, Austria) was applied according to the manufacturer’s protocol (https://bit.ly/3fTINGj). The quality and quantity of the library QC was performed by using High Sensitivity DNA1000 ScreenTape system with 2200 Tapestation (Agilent Technologies, Santa Clara, CA, USA) (https://bit.ly/3lZ7wgp) and dsDNA HS Assay Kit with Qubit 3.0 Fluorometer (Thermo Fisher Scientific, Waltham, MA, USA) (https://bit.ly/3Az1vLf), respectively. Pooled libraries were diluted to 2 pM for 1 × 86 bp single-end sequencing with 75-cycle High Output v2 Kit on the NextSeq 550 Sequencing System (Illumina, San Diego, CA, USA) according to the manufacturer’s protocol (https://bit.ly/39akWOE).

### Quantification and differential expression analysis

RNA-seq data analysis was partially performed using the Galaxy platform^113^. The single-end, raw Illumina NextSeq 550 read lanes were concatenated per samples using a Concatenate datasets tail-to-head tool v1.0.0 (Galaxy). Low-quality and adaptor sequences in the raw data were eliminated using the Trimmomatic v.0.36.5 tool^114^ with the following parameters: SLIDINGWINDOW:4:20; MINLEN:20. Both of the raw and trimmed reads were quality checked and analyzed by FastQC v0.72 (https://www.bioinformatics.babraham.ac.uk/projects/fastqc/). The trimmed and quality filtered reads were mapped to the *C. annuum* reference genome (https://www.ncbi.nlm.nih.gov/assembly/GCF_000710875.1_Pepper_Zunla_1_Ref_v1.0/) using an RNA STAR v2.7.2b aligner^115^ with single-sample 2-pass mapping mode (–twopassMode Basic). The properly mapped reads were counted from the alignment files by the featureCounts v1.6.4 tool^116^, then the DESeq2 v2.11.40.6 tool^117^ was used to determine differentially expressed genes (DEGs) from the count tables. Only the gene expression records which passed the following criterion were used for further analysis: *p* ≤ 0.01 and − 1 ≥ log2 fold change (FC) ≥ 1. RNA-seq raw data were deposited at ArrayExpress (E-MTAB-10769) and European Nucleotide Archive (PRJEB46507).

In a separate bioinformatical analysis with the above methods we established the lists of pepper genes that were inducible by 5 mM sodium salicylate, 100 μM methyl-jasmonate, 5 mM ethephon (ethylene precursor) and 100 μM abscisic acid (ABA) by using the publicly available raw gene expression data of Lee et al. (2020)^42^ from the Sequence Read Archive (SRP265260). We aligned these hormone-inducibility data with the ObPV- and PMMoV-inducibility of pepper genes obtained by our RNA-Seq analyses.

### Functional annotation

The functional annotation of DEGs were basically performed in 3-way approaches. To determine functional domains from DEGs, their associated amino acid sequences were scanned with the Hidden Markov Model (HMM)-based HMMER v3.3.1 software package^118^ using Pfam-A v32.1 HMM profiles (ftp://ftp.ebi.ac.uk). For Gene Ontology (GO) analysis an up-to-date ontology database (http://geneontology.org/docs/download-ontology; goslim_generic and goslim_plant) and custom-made gene annotation dataset were necessary based on ontology information of the Universal Protein Resource (ftp://ftp.uniprot.org/pub/databases/uniprot/previous_releases/release-2019_04/). The enrichment analysis was performed with the BiNGO v3.0.3 plug-in^119^ to the Cytoscape v3.7.2 software^120^. The over-represented GO terms were determined using a hypergeometric statistical test with Benjamin & Hochberg False Discovery Rate (FDR) correction at *p* ≤ 0.05 significance level. The Wget software package (https://www.gnu.org/software/wget/) was used to retrieve the corresponding datasets from the KEGG knowledgebase^43^, including functional annotations, metabolic pathway classifications and nucleotide/protein sequences. All the annotation data from different sources were combined in a single document (Supplementary Table S2).

### Real-time, quantitative RT-PCR measurements

Quantitative, real-time RT-PCR (qPCR) assays were conducted with the same total RNA samples that were used for RNA-Seq studies. Reverse transcription (RT) of 1.5 µg total RNA was carried out in a total volume of 20 µl with a RevertAid H Minus First Strand cDNA Synthesis kit (Thermo Fisher, Waltham, MA, USA) using an oligo(dT)18 primer. In the case of virus multiplication measurements, RT of viral RNAs were carried out by using the reverse primers of the viral movement protein (MP) or coat protein (CP) gene-specific primer pairs (Supplementary Table S7) instead of an oligo(dT)18 primer. Real-time PCR analyses were carried out by using a qPCRBIO SyGreen Blue Mix Separate-Rox kit (PCR Biosystems, London, UK). Reaction mixtures contained 7.5 µl SyGreen Blue Mix, 0.25 µM of each primer (Supplementary Table S7) and 1 µl of tenfold diluted cDNA template in a final volume of 15 µl. For the thermal amplification a CFX 96 Touch™ Real-Time PCR Detection System (Bio-Rad Laboratories, Hercules, CA, USA) was used. Cycling parameters were: initial denaturation at 95 °C for 3 min, then 40 cycles of 95 °C for 10 s, 30 s at specific annealing temperatures (see Supplementary Table S7 for each primer pair), and 72 °C for 30 s, followed by a cDNA dissociation program from 65 to 95 °C to create melting curves. Serial dilutions of the pooled cDNA samples were used to generate standard curves to obtain the amplification efficiencies of PCR reactions. For control housekeeping gene a pepper gene encoding ubiquitin-conjugating enzyme 3 (*CaUBC3*) was selected (Supplementary Table S7). Our preliminary evaluation indicated that expression of *CaUBC3* displays little variation across all samples tested, which is in line with earlier results^121^. Since all PCR amplification efficiencies were near 100%, the expression values of target genes were first normalized to *CaUBC3* and then the obtained relative expression levels were compared to mock-inoculated values, separately for each sampling time point, according to the method of Livak and Schmittgen (2001)^97^. All RT-qPCR assays were carried out in three independent parallel experiments.

### Pepper protein interactome analysis

Our knowledge about interactions between pepper proteins is still rudimentary, therefore the well characterized *Arabidopsis thaliana* interactome datasets by BioGRID^98^ version 3.5.138 were called for help in our analysis. BLASTP similarity searches were performed to identify the best Arabidopsis/pepper orthologs. In the first step, protein sequences of pepper DEGs were searched against the Arabidopsis proteins, then the search was made in the opposite direction, as well. Those pepper proteins, which proved to be similar to Arabidopsis proteins in both directions were considered as genuine orthologs and these pepper proteins were used to replace Arabidopsis proteins in the Arabidopsis interactome. The protein–protein interaction network topology was designed and visualized with the yEd Graph Editor software version 3.21.1 (https://www.yworks.com/products/yed).

## Supplementary Information

Supplementary Table S1.

Supplementary Table S2.

Supplementary Table S3.

Supplementary Table S4.

Supplementary Table S5.

Supplementary Table S6.

Supplementary Table S7.
